# Efficacy and safety of intralesional corticosteroids alone or in combination with other treatments for the management of keloids: a systematic review and meta-analysis

**DOI:** 10.3389/fmed.2026.1930913

**Published:** 2026-10-26

**Authors:** Naif Abed Alshamari, Alanoud AlDaej, Asmaa Yaser Mallisho, Reemana Waleed Alsudais, Moudi Nayef Alhajraf, Danah Mohammad, Ahmed Turki Al-Turki, Mousa Amer Alshehri, Reem T. Alshammari, Abdullah N. AlBarrak

**Affiliations:** 1 College of Medicine, Alfaisal University, Riyadh, Saudi Arabia; 2 College of Pharmacy, Alfaisal University, Riyadh, Saudi Arabia; 3 Primary Health Care, Ministry of Health, Riyadh, Saudi Arabia; 4 Jahra Hospital, Ministry of Health, Al Jahra, Kuwait; 5 Fawanya Hospital, Ministry of Health, Al Fawanya, Kuwait; 6 College of Medicine, King Khalid University, Abha, Saudi Arabia; 7 Aseer Central Hospital, Ministry of Health, Abha, Saudi Arabia; 8 Dammam Medical Complex, Dammam, Saudi Arabia; 9 King Fahad Medical City, Riyadh, Saudi Arabia

**Keywords:** combination, corticosteroids, intralesional, keloid, monotherapy

## Abstract

**Background:**

Keloids continue to present a therapeutic challenge despite several proposed treatment modalities, and there is a lack of current evidence-based guidelines to inform disease management. Therefore, we conducted this systematic review and meta-analysis to compare intralesional corticosteroid monotherapy against combination therapies containing intralesional corticosteroids regarding their efficacy and safety.

**Methods:**

We searched PubMed, Scopus, and Web of Science using the Medical Subject Headings (MeSH) terms with the following keywords: “Corticosteroids” AND “Keloid” AND “Intralesional” from inception until May 2026 to identify the studies included in this systematic review and meta-analysis. The screening process was conducted based on inclusion and exclusion criteria established using the PICOS criteria as follows: P (population): patients with keloids; I (intervention): a combination of intralesional corticosteroids and any other therapy or drug; C (comparison): intralesional corticosteroids alone; O (outcomes): any efficacy and safety outcome; and S (study design): randomized controlled trials, non-randomized trials, and observational cohort studies.

**Results:**

A total of 20 studies were included in the current systematic review and meta-analysis, comprising 15 randomized controlled trials, 2 non-randomized controlled trials, and 3 cohort studies. The analysis of each outcome included only a subset of the included studies, as not all studies reported all outcomes. The analysis favored combination treatment over intralesional corticosteroids alone regarding clinical improvement or efficacy, with an odds ratio of 2.71 (95% CI: 1.8, 4.09; *p* < 0.00001, I^2^ = 17%). Combination therapy was observed to be associated with greater baseline improvement in clinical signs of keloids, such as pain, itching, pigmentation, and height or thickness, with a mean difference (MD) of 0.67 (95% CI: 0.27, 1.06, *p* = 0.0009, *I*^2^ = 69%), a standardized mean difference of 0.41 (95% CI: 0.06, 0.77, *p* = 0.02, I^2^ = 0%), an MD of 0.66 (95% CI: 0.4, 0.92, *p* < 0.00001, *I*^2^ = 33%), and an MD of 0.15 (95% CI: 0.06, 0.23, *p* = 0.0005, *I*^2^ = 85%), respectively, with *I*^2^ = 85%, *p* < 0.00001.

**Conclusion:**

Combination therapies with intralesional corticosteroids showed greater efficacy and safety than monotherapy across various outcomes. Subgroup analysis indicates that effects vary by co-intervention: corticosteroids (CS) plus 5-fluorouracil (5-FU) had favorable efficacy, adverse events, and height outcomes; Vancouver Scar Scale change favored CS plus excision, 5-FU, and platelet-rich plasma. Significant subgroup differences, heterogeneity, and limited data warrant cautious interpretation. Clinical and methodological differences, treatment variations, and inclusion of non-randomized studies limit certainty and generalizability. Evidence does not universally favor combination therapies over monotherapy. Among adjuncts, 5-FU combinations show consistent benefit, but more trials are needed to identify the best regimen.

**Systematic Review Registration:**

https://www.crd.york.ac.uk/PROSPERO/view/CRD420261439889, identifier CRD420261439889.

## 1 Introduction

Keloid lesions typically arise from abnormal wound healing after skin injury and present as nodular lesions that extend beyond the original injury margins. Keloids negatively affect patients' quality of life due to pain, itching, and their disfiguring appearance, which generally correlates with size (1). Furthermore, keloids demonstrate a significant recurrence rate following treatment, posing a substantial challenge for effective treatment. Certain anatomical sites exhibit a heightened propensity for keloid formation, notably the ear, anterior thorax, upper back, shoulders, and upper extremities (2). In addition, specific anatomical locations and treatment methods are associated with a higher propensity for recurrence (3).

Various mechanisms have been proposed for the pathophysiology of keloids. Proinflammatory cytokines IL-6 and IL-8 have been shown to intensify scarring, while a decrease in the anti-inflammatory cytokine IL-10 also increases scarring (4). Keloidal fibroblasts and inflammatory cells contribute to keloid formation by disrupting normal collagen metabolism. Keloids are characterized by an increased ratio of type 1 to type 3 collagen deposited in a disordered fashion, along with enhanced fibroblast proliferation rates and heightened sensitivity to growth stimuli. Differences in growth factor production may arise from epithelial–mesenchymal interactions, maintenance of embryonic proliferative pathways, or keloidal tissue hypoxia. Tissue tension is recognized as a mechanical stress that activates fibroblast activity and promotes collagen formation. Certain inherited human leukocyte antigen subtypes are associated with keloids, suggesting that an abnormal immune response to skin injury may play a role in their development. It has been postulated that dermal injury induces an immunological response to sebum, leading to cytokine release that facilitates mast cell infiltration and fibroblast activity, thereby explaining the propensity for keloids to form in areas with a higher density of pilosebaceous units (5, 6).

Current therapeutic techniques include intralesional and topical treatments, surgical interventions, radiation, and laser therapies. Intralesional corticosteroids are a key therapeutic opinion, but alternative injectables include bleomycin, 5-fluorouracil (5-FU), botulinum toxin type A, verapamil, avotermin, IL-10, mannose-6-phosphate, and insulin. Topical therapies include imiquimod and mitomycin C. Surgical excisions are often supplemented with adjuvant pharmacotherapies, and there is ongoing progress in keloid excision and wound closure methodologies. Radiation therapies include external-beam radiation and interstitial brachytherapy administered at low or high dosage rates (7–9). Pulsed dye laser (PDL), cryotherapy, pressure dressings, over-the-counter silicone sheets, and topical vitamin E preparations are commonly utilized. Despite several proposed treatment modalities, keloids continue to represent a therapeutic challenge, and there is a lack of current evidence-based guidelines to inform disease management. Although several adjunctive therapies have been evaluated in combination with intralesional corticosteroids, the evidence remains fragmented across individual treatment modalities, and the comparative efficacy and safety of combination therapy as a broader treatment strategy remain incompletely characterized. In particular, uncertainty persists regarding whether the potential benefits of combination treatment are consistent across different clinical outcomes and adjunctive modalities. Therefore, we conducted this systematic review and meta-analysis to compare intralesional corticosteroid monotherapy with combinations containing intralesional corticosteroids in terms of efficacy and safety.

## 2 Methods

Following the Cochrane Handbook of Systematic Reviews of Interventions (10) and the Preferred Reporting Items for Systematic Reviews and Meta-Analyses (PRISMA) guidelines (11), we conducted this systematic review and meta-analysis. The protocol was prospectively registered and is publicly available through the PROSPERO database (CRD420261439889).

### 2.1 Database search

We searched PubMed, Scopus, and Web of Science using the Medical Subject Headings (MeSH) terms with the following keywords: “Corticosteroids” AND “Keloid” AND “Intralesional” from inception until May 2026 to identify the articles included in this systematic review and meta-analysis (Supplementary Table S1).

### 2.2 Screening

The resulting articles from the search process were uploaded to Rayyan (12) to remove duplicates and conduct the title and abstract screening. Eligible articles from this step proceeded to full-text screening to determine their eligibility for inclusion in the systematic review and meta-analysis. Title and abstract screening and subsequent full-text assessment were conducted independently by two reviewers. Disagreements regarding study eligibility were resolved through discussion and consensus, with involvement of a senior reviewer when consensus could not initially be reached.

### 2.3 Inclusion and exclusion criteria

The screening process was conducted based on inclusion and exclusion criteria established upon PICOS criteria as follows: P (population): patients with keloid; I (intervention): combination of intralesional corticosteroids and any other therapy or drug; C (comparison): intralesional corticosteroids alone; O (outcomes): any efficacy and safety outcome; and S (study design): randomized controlled trials (RCTs), non-randomized trials, and observational cohort studies. We excluded studies that did not include a combination or that included one not involving intralesional corticosteroids and those that did not compare the combination with intralesional corticosteroids alone. We also excluded reviews and case reports.

### 2.4 Quality and risk of bias assessment

For the included observational cohort studies, we used the Newcastle–Ottawa Scale (NOS) tool provided by Cochrane to assess quality. A score of 0–3 was considered low quality, 4–6 was considered moderate quality, and 7–9 was considered high quality (13). For RCTs, we used the Cochrane Risk of Bias 2 (Rob-2) tool to assess bias in the included studies (14). For non-randomized trials, we used the Risk Of Bias In Non-randomized Studies—of Interventions (ROBINS-I) for the risk-of-bias assessment (15). This process was conducted independently by two reviewers. Disagreements were resolved through discussion and consensus, with involvement of a senior reviewer when consensus could not initially be reached.

### 2.5 Data extraction

Using Microsoft Excel spreadsheets, we conducted data extraction. We extracted baseline data from the included studies, including sample size, patient age and gender, definition of clinical improvement, combination therapy versus monotherapy, and follow-up duration. We also extracted outcome data, including clinical efficacy or improvement, adverse events, recurrence, baseline and post-treatment values of pain, itching, pigmentation, thickness or height, Vancouver Scar Scale (VSS) scores, and Patient and Observer Scar Assessment Scale (POSAS) scores. This process was conducted independently by two reviewers. Disagreements were resolved through discussion and consensus, with involvement of a senior reviewer when consensus could not initially be reached.

### 2.6 Statistical analysis

We calculated improvements for all outcomes using the Meta-Analysis Accelerator (https://ma-accelerator.com/) with the baseline and post-treatment values. For continuous outcomes reported as changes from baseline, change scores were calculated as post-treatment minus baseline values where necessary. When the standard deviation of the change score was not directly reported, it was calculated using the standard formula incorporating the baseline and post-treatment standard deviations and the assumed correlation between baseline and post-treatment measurements, as specified by the provided software, which is based on the Cochrane Guidelines. We used Review Manager software (16) for all statistical analyses. We calculated the pooled odds ratio (OR) for dichotomous data and the pooled mean difference (MD) or standardized mean difference (SMD) for continuous data. We performed a subgroup analysis to divide the combination therapies based on whether intralesional corticosteroids were used. Therapies mentioned in two or more studies were included in a subgroup. Therapies mentioned in only one study were categorized as “other treatments,” which included pentoxifylline, cryotherapy, onion extract gel, and hyaluronidase. We used a fixed-effects model for homogeneous data (which were found to be homogeneous and showed no significant heterogeneity after applying the random-effects model) and a random-effects model for heterogeneous data. A confidence interval (CI) of 95% was used, and a *p*-value was considered significant if it was equal to or less than 0.05. Heterogeneity was assessed using *I*^2^ and the *p*-value.

### 2.7 Certainty of evidence assessment

The certainty of evidence for the main outcomes was assessed using the Grading of Recommendations Assessment, Development and Evaluation (GRADE) framework. Certainty was evaluated separately for each outcome across five domains: risk of bias, inconsistency, indirectness, imprecision, and publication bias. Evidence from randomized controlled trials was initially considered high certainty, whereas evidence from non-randomized and observational studies was initially considered low certainty. The certainty rating was subsequently downgraded when important concerns were identified in one or more GRADE domains. Risk of bias was assessed using the study-level risk-of-bias assessments. Inconsistency was assessed based on the magnitude of statistical heterogeneity, the direction and overlap of individual study estimates, and the clinical plausibility of observed differences. Indirectness was assessed with respect to the study population, intervention and comparator, outcomes, and applicability to the review question. Imprecision was considered according to the width of the confidence intervals, the number of participants and events, and whether the confidence interval included both a clinically important benefit and little or no effect. Potential publication bias was assessed considering the number of contributing studies, study size, selective outcome reporting, and the feasibility of formal assessment of small-study effects. For pooled outcomes across different study designs, the certainty of the evidence was interpreted conservatively, taking into account the contributions of non-randomized and observational studies. The final certainty of evidence was categorized as high, moderate, low, or very low.

## 3 Results

### 3.1 Database searching and screening

After searching the databases, we retrieved 762 articles, including 259 duplicates. We conducted title and abstract screening for the remaining 503 articles. We excluded 472 articles and conducted full-text screening for the remaining 31 articles. After exclusion of irrelevant studies, a total of 20 studies were included in the current systematic review and meta-analysis (1, 17–35) (Figure 1).

**Figure 1:**
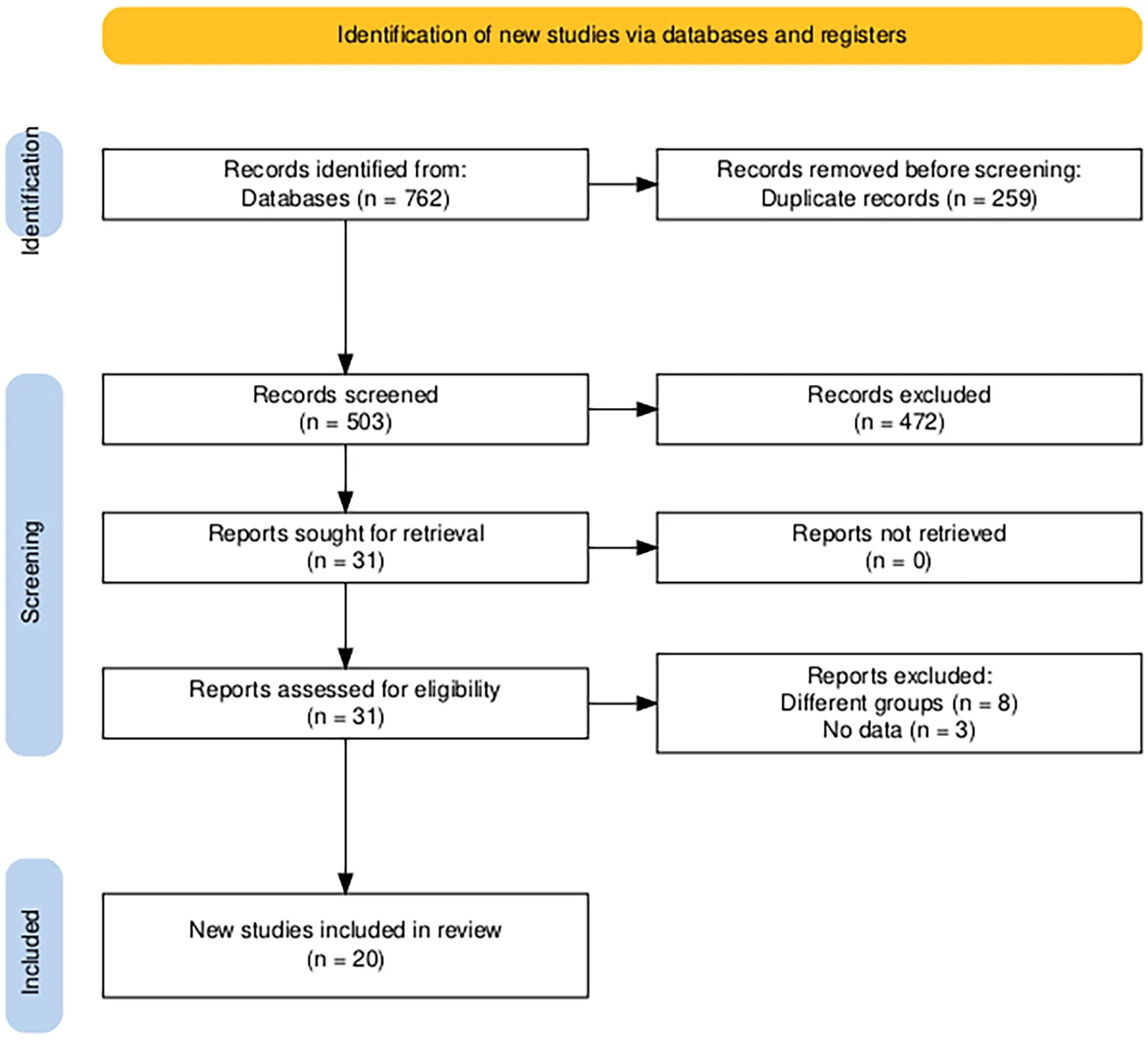
PRISMA flow diagram of the search and screening processes.

### 3.2 Quality and risk of bias assessment

According to the NOS, the three cohort studies were deemed to have high quality (Table 1). Regarding the included RCTs and non-randomized trials, they were observed to exhibit a low risk of bias (Figures 2, 3).

**Table 1:** Quality assessment of cohort studies using the NOS scale.

Study ID	Selection (max 4)	Comparability (max 2)	Outcome (max 3)	Total (max 9)
Yıldız (18)	⋆⋆⋆	⋆⋆	⋆⋆⋆	⋆⋆⋆⋆⋆⋆⋆⋆
Alexander (21)	⋆ ⋆ ⋆	⋆⋆	⋆⋆⋆	⋆⋆⋆⋆⋆⋆⋆⋆
Shin (32)	⋆ ⋆ ⋆	⋆	⋆⋆⋆	⋆⋆⋆⋆⋆⋆⋆

**Figure 2:**
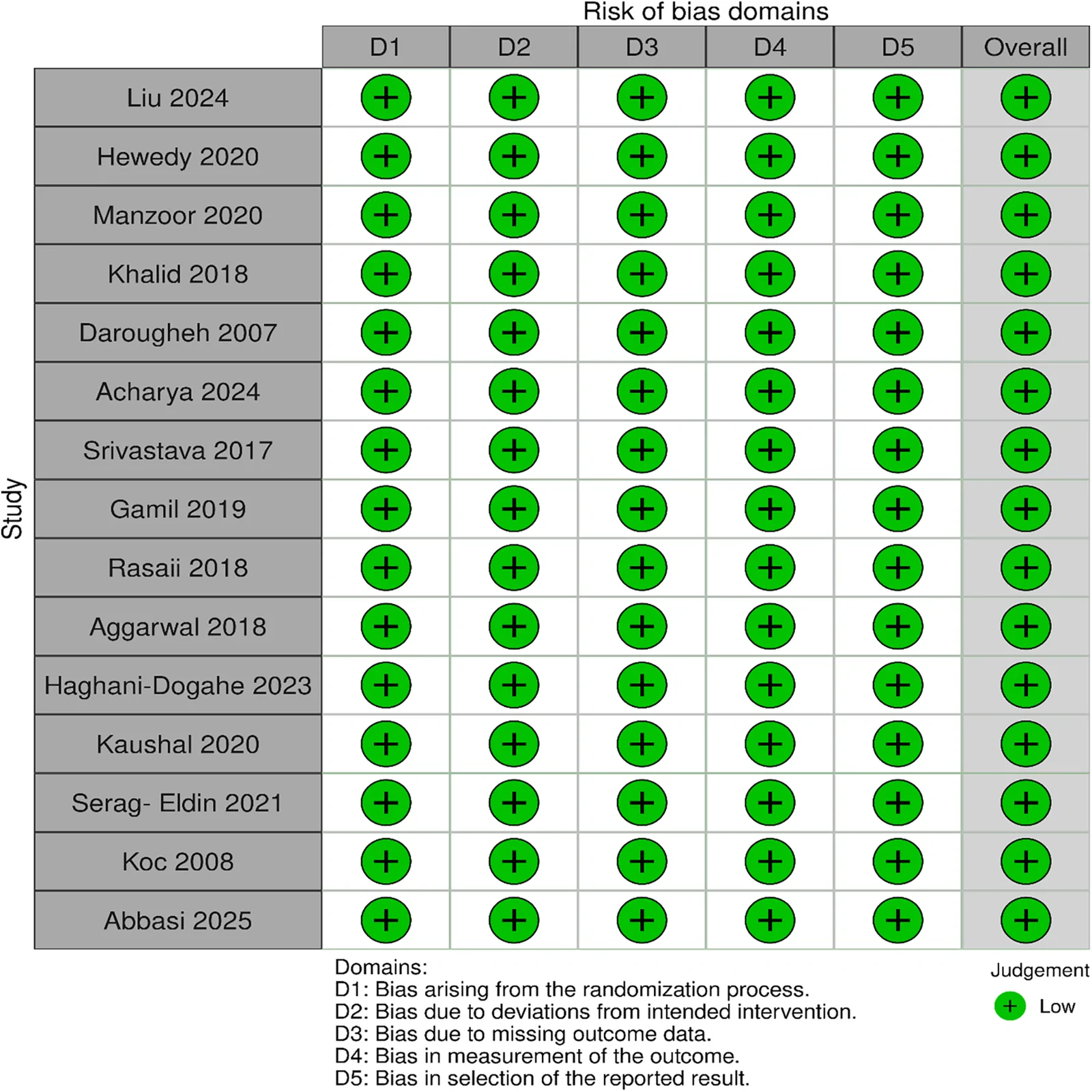
Risk-of-bias assessment of the included randomized controlled trials using the Rob-2 tool.

**Figure 3:**
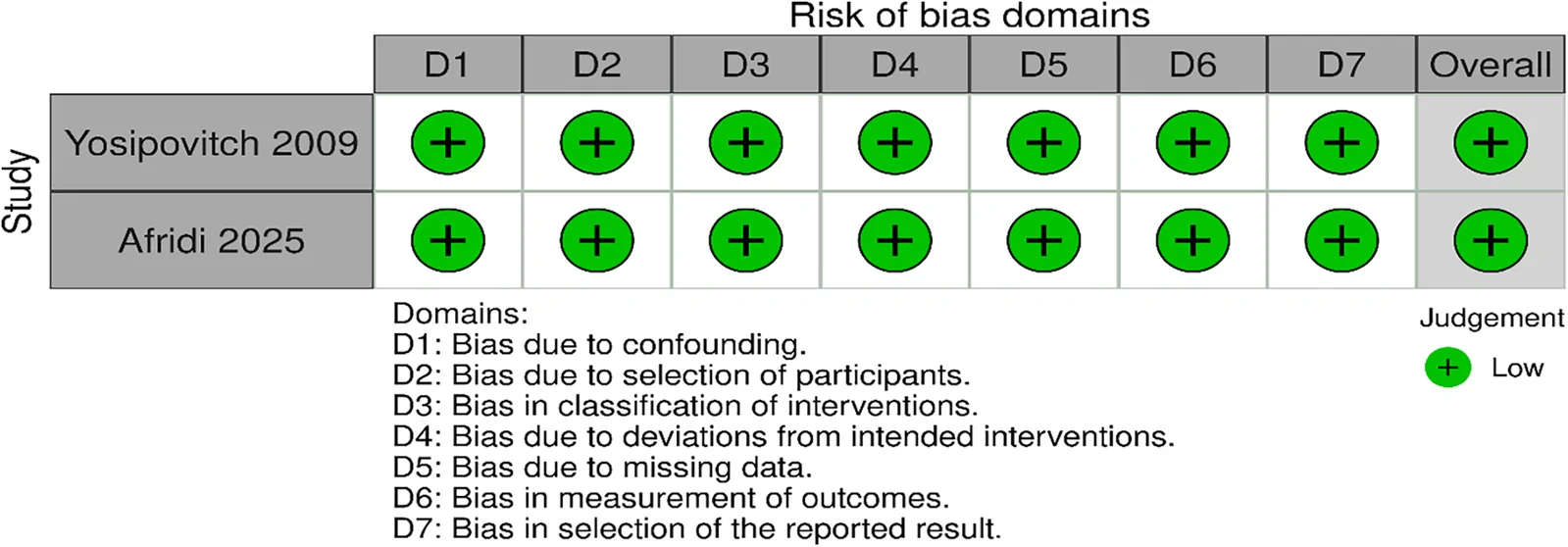
Risk-of-bias assessment of the included non-randomized trials using the ROBINS-I tool.

### 3.3 Baseline characteristics

A total of 15 RCTs, 2 non-randomized trials, and 3 cohort studies were included in the current systematic review and meta-analysis. Combination therapies varied depending on the drug or therapy used in conjunction with intralesional corticosteroids, including 5-FU, verapamil, Botox, cryotherapy, excision, laser therapy, pentoxifylline, onion extract, radiofrequency, and hyaluronidase. The definition of clinical improvement differed between the studies. Clinical improvement/efficacy was analyzed as a dichotomous outcome according to the definition reported by each study. Although the specific criteria varied, they represented the common clinical construct of a favorable treatment response. Generally, they reflected substantial reduction in keloid height or size, flattening or softening of the lesion, improvement in validated scar-assessment scores, or a combination of morphological and symptomatic improvement. Participants meeting the original study-defined criterion were classified as having achieved clinical improvement, whereas those not meeting that criterion were classified as not having achieved improvement. Liu et al. defined improvement as disappearance or substantial alleviation of symptoms, together with marked softening and flattening of the keloid, and absence of recurrence during follow-up. Manzoor et al. defined improvement as 51%–100% improvement based on flattening and reduction in lesion size, whereas Khalid et al. used a >50% reduction in initial scar height. Acharya et al. defined improvement as a ≥50% reduction in initial height or >50% subjective improvement reported by the patient or observer. Aggarwal et al. used a reduction of keloid height to ≤1 mm, Kaushal et al. used a >75% reduction in POSAS score, and Abbasi et al. defined improvement as a >75% reduction in the VSS score from baseline (Table 2).

**Table 2:** Baseline characteristics of the included studies.

Study ID	Design	Sample size		Age, mean (SD)		Male, *n* (%)		Combination	Monotherapy	Follow-up	Clinical improvement definition
				Combination	Monotherapy	Combination	Monotherapy				
Yıldız (18)	Cohort	40	20	36 (11)	36 (9.5), 29 (7.25)	12 (60%)	14 (70), 10 (50)	Surgical excision and TAC, or PRP-surgical excision and TAC	Intralesional TAC	5 years	
Liu (25)	RCT	50	50	NR	NR	NR	NR	Intralesional betamethasone + single-hole punch excision	Intralesional betamethasone	1 year	The pain disappears, the keloid is completely softened and flattened, and it is soft and has no indurations, and there is no recurrence after 1 year of follow-up after treatment; the symptoms, such as itching, disappeared or were significantly alleviated, and 60%–70% of the keloid area softened and flattened, and there was no recurrence after 1 year of follow-up
Hewedy (17)	RCT	20	20	29.05 (12.32)	23.71 (8.41)	11 (55)	9 (45)	Intralesional TAC + PRP	Intralesional TAC	3 months	
Manzoor (24)	RCT	30	30	28.17 (8.69)	29.10 (9.47)	NR	NR	Intralesional TAC + 5-FU	Intralesional TAC	6 months	Defined as 51–100% improvement (flattening and decrease in size of lesion)
Khalid (19)	RCT	57	51	27.67 (9.48)	31.22 (12.559)	24 (42.1)	22 (43%)	Intralesional TAC + 5-FU	Intralesional TAC	3 months	Defined as >50% reduction in initial scar height)
Darougheh (26)	RCT	20	20	25.2 (11.9)	23.4 (8)	NR	NR	Intralesional TAC + 5-FU	Intralesional TAC	3 months	
Acharya (33)	RCT	32	31	NR	NR	NR	NR	Intralesional TAC + 5-FU	Intralesional TAC	3 months	A 50% or more reduction in the initial height or more than 50% subjective improvement as reported by the patient or observer at 12 weeks
Srivastava (1)	RCT	20	20	29.9 (10.19)	26.35 (6.11)	8 (40)	8 (40)	Intralesional TAC+5-FU	Intralesional TAC	6 months	
Gamil (23)	RCT	24	26	27.8 (6)	28.4 (6.5)	16 (66.7)	18 (69.2)	Intralesional TAC + Botox	Intralesional TAC	6 months	
Rasaii (28)	RCT	23	23	23.3 (1.2)		11 (48)		Intralesional TAC + Botox	Intralesional TAC	NR	
Yosipovitch (20)	Non-randomized controlled study	10	10	25.9 (9.9)	25.9 (9.9)	9 (90)	9 (9)	Intralesional TAC + cryotherapy	Intralesional TAC	8 months	
Alexander (21)	Cohort	25	25	NR	NR	18 (72)	20 (80)	Intralesional TAC + Fractional CO_2_ laser	Intralesional TAC	NR	
Shin (32)	Cohort	17	21	37.23 (10.75)	41.38 (11)	9 (52.9)	8 (38.1)	Intralesional TAC + non-ablative fractional laser	Intralesional TAC	6 months	
Aggarwal (27)	RCT	32	16	30.41		NR	NR	Intralesional TAC + hyaluronidase, or intralesional TAC + intralesional radiofrequency	Intralesional TAC	NR	reduction in keloid height to 1 mm or less
Haghani-Dogahe (30)	RCT	16	16	35.5 (8.27)	36.69 (8.95)	9 (56.3)	9 (56.3)	Intralesional TAC + intralesional verapamil hydrochloride	Intralesional TAC	3 months	
Kaushal (29)	RCT	30	30	32.2	34.93	29 (48.3)		Intralesional TAC + intralesional radiofrequency	Intralesional TAC	8 months	>75% reduction in the POSAS score
Serag-Eldin (22)	RCT	10	10	25.9 (11.2)	19 (13.8)	4 (40)	6 (60)	Intralesional TAC + pentoxifylline	Intralesional TAC	NR	
Koc (31)	RCT	14	13	28.1 (11.7)		17 (63)		Intralesional TAC + onion extract gel	Intralesional TAC	NR	
Afridi (35)	Non-randomized controlled study	30	30	28.4 (9.239)	27.37 (5.968)	NR	NR	Intralesional TAC + PRP	Intralesional TAC	NR	
Abbasi (34)	RCT	38	38	30.29 (6.50)	30.29 (5.80)	26 (68.4)	25 (65.8)	Intralesional TAC + cryotherapy	Intralesional TAC	3 months	Defined as a >75% reduction in the VSS score from baseline at the end of the treatment

RCT, randomized controlled trial; SD, standard deviation; NR, not reported; PRP, platelet-rich plasma; TAC, triamcinolone acetonide.

### 3.4 Statistical analysis

The analysis favored combination treatments over intralesional corticosteroids alone for clinical improvement or efficacy, with an OR of 2.71 (95% CI: 1.8, 4.09; *p* < 0.00001) and *I*^2^ = 17%, *p* = 0.3. The effect was tested across subgroups, showing significant differences in the combinations of intralesional corticosteroids with surgical excision and 5-FU but no differences with combinations involving radiofrequency or other treatments such as hyaluronidase (Figure 4).

**Figure 4:**
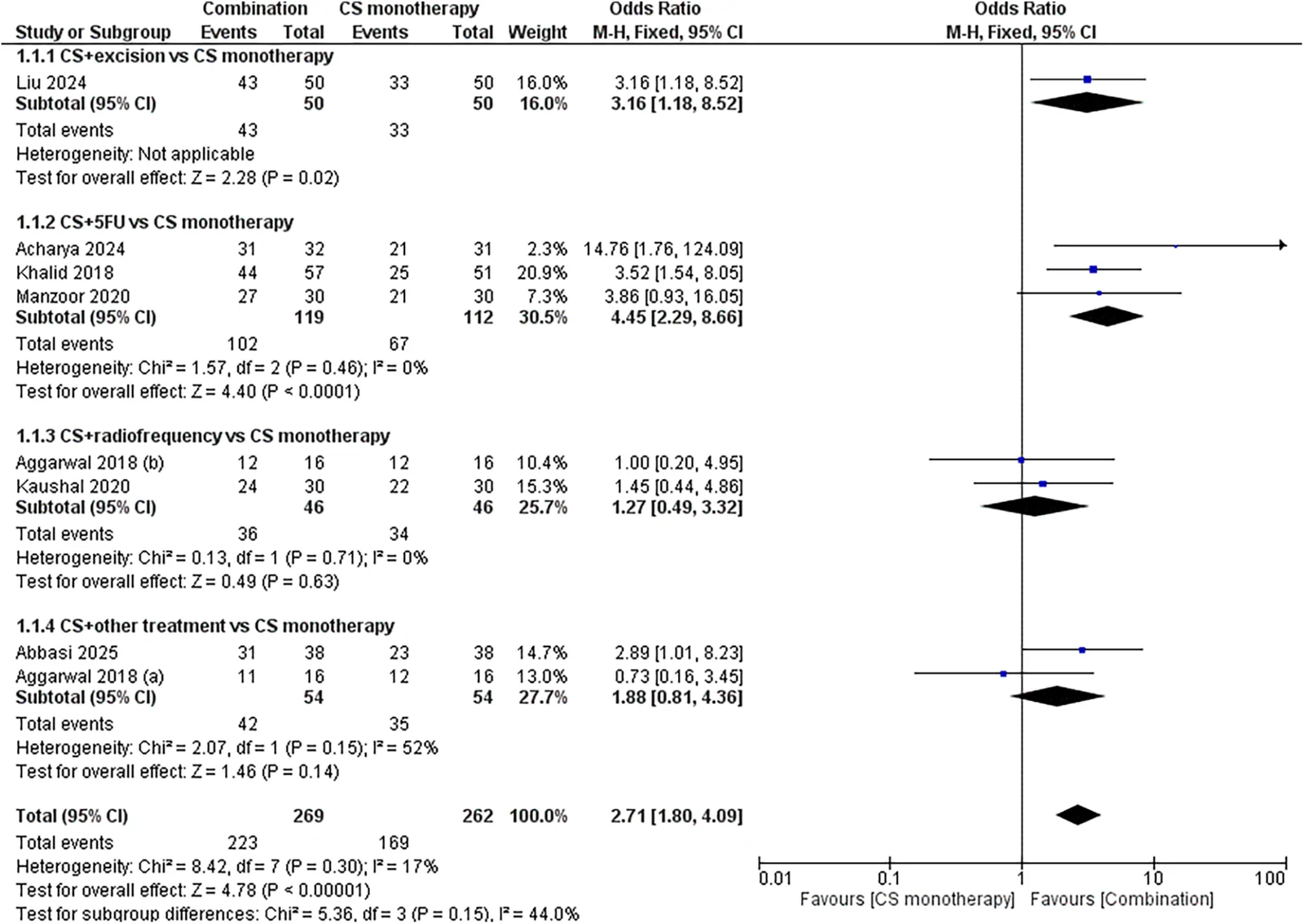
Comparison between combination and monotherapy of intralesional corticosteroids regarding clinical improvement (efficacy), sub-grouped by the therapy used in the combination.

Combination therapy was associated with greater improvement from baseline in clinical signs of keloids, such as pain, itching, pigmentation, and height or thickness. The MD for pain was 0.67 (95% CI: 0.27, 1.06; *p* = 0.0009) with *I*^2^ = 69%, *p* = 0.003. For itching, the SMD was 0.41 (95% CI: 0.06, 0.77; *p* = 0.02) with *I*^2^ = 0%. For pigmentation, the MD was 0.66 (95% CI: 0.4, 0.92; *p* < 0.00001) with *I*^2^ = 33%, *p* = 0.22. For height or thickness, the MD was 0.15 (95% CI: 0.06, 0.23; *p* = 0.0005) with *I*^2^ = 85%, *p* < 0.00001. However, in the height subgroup, the combination containing 5-FU was the only combination associated with more improvement in height compared to intralesional corticosteroid monotherapy; other combinations showed no significant difference (Figures 5–8).

**Figure 5:**
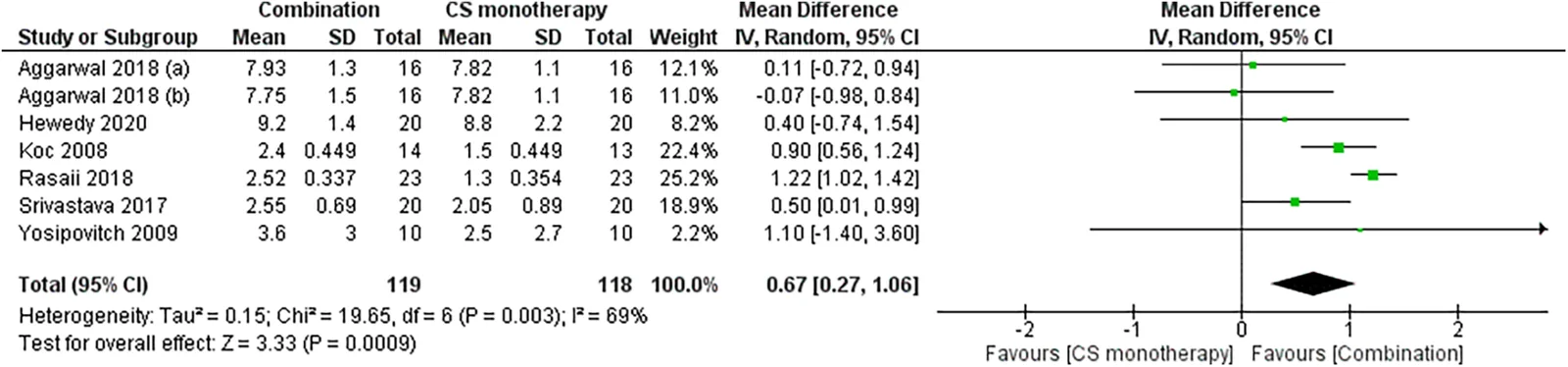
Comparison between combination therapy and monotherapy of intralesional corticosteroids regarding improvement of pain.

**Figure 6:**
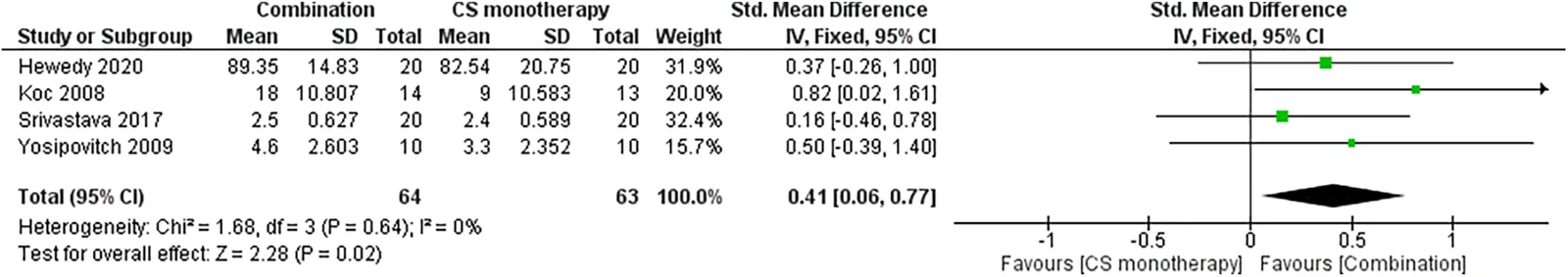
Comparison between combination therapy and monotherapy of intralesional corticosteroids regarding improvement of itching.

**Figure 7:**
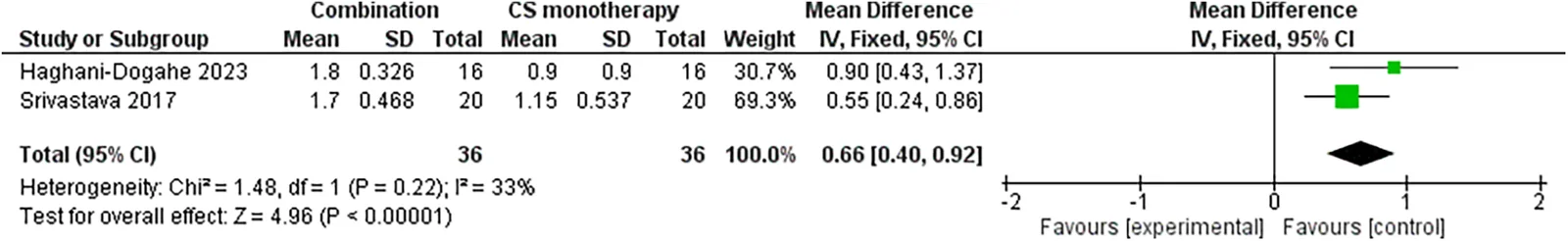
Comparison between combination therapy and monotherapy of intralesional corticosteroids regarding improvement of pigmentation.

**Figure 8:**
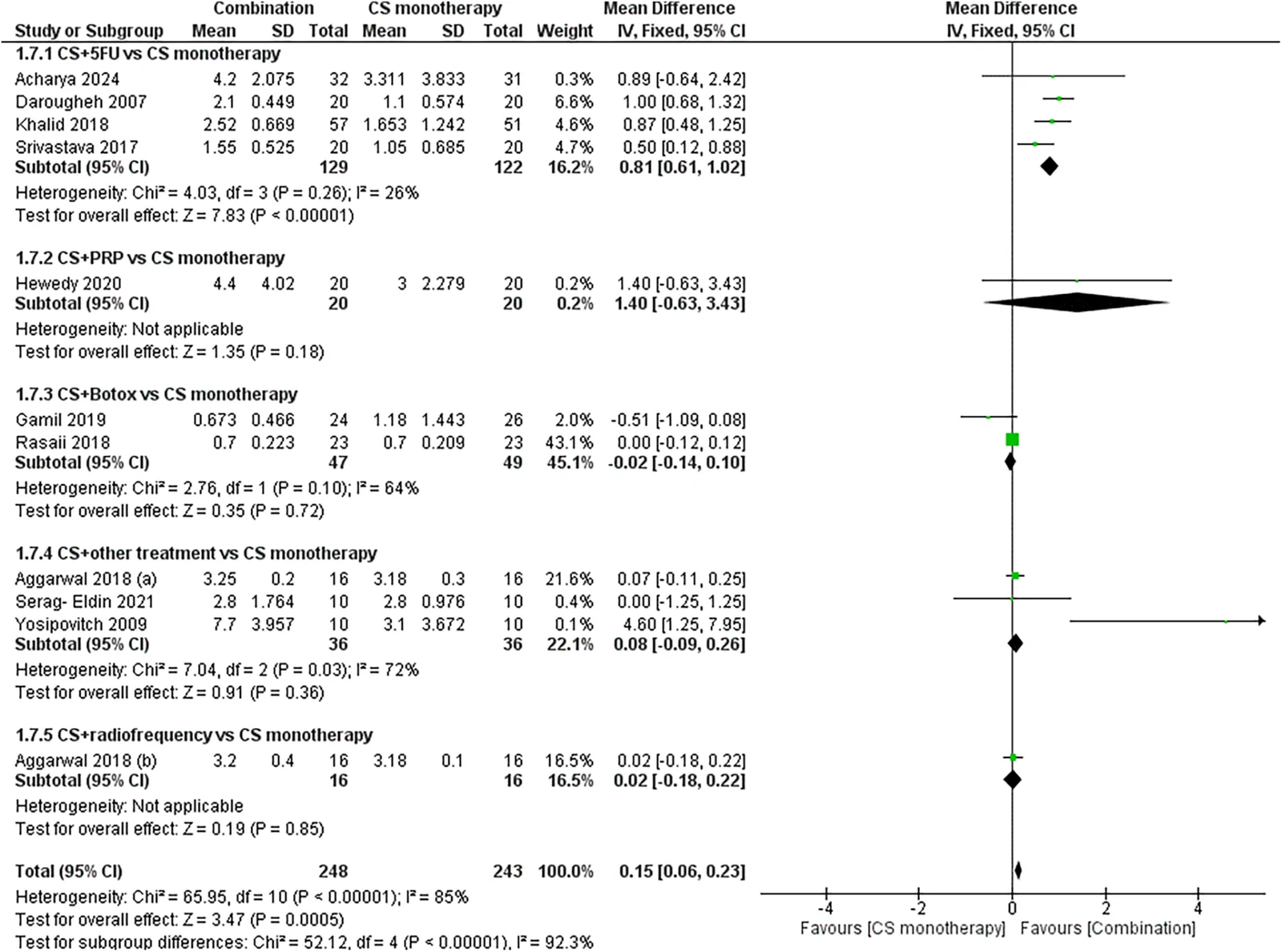
Comparison between combination therapy and monotherapy of intralesional corticosteroids regarding improvement in height, sub-grouped by the therapy used in the combination.

Regarding the VSS change from baseline, combination therapy was associated with a greater change than monotherapy, with a MD of 1.07 (95% CI: 0.13, 2.02; *p* = 0.03). Also, combination therapy was significantly associated with better improvement in the POSAS score, with an MD of 8.02 (95% CI: 6.22, 9.82; *p* < 0.00001) and *I*^2^ = 0% (Figures 9,10).

**Figure 9:**
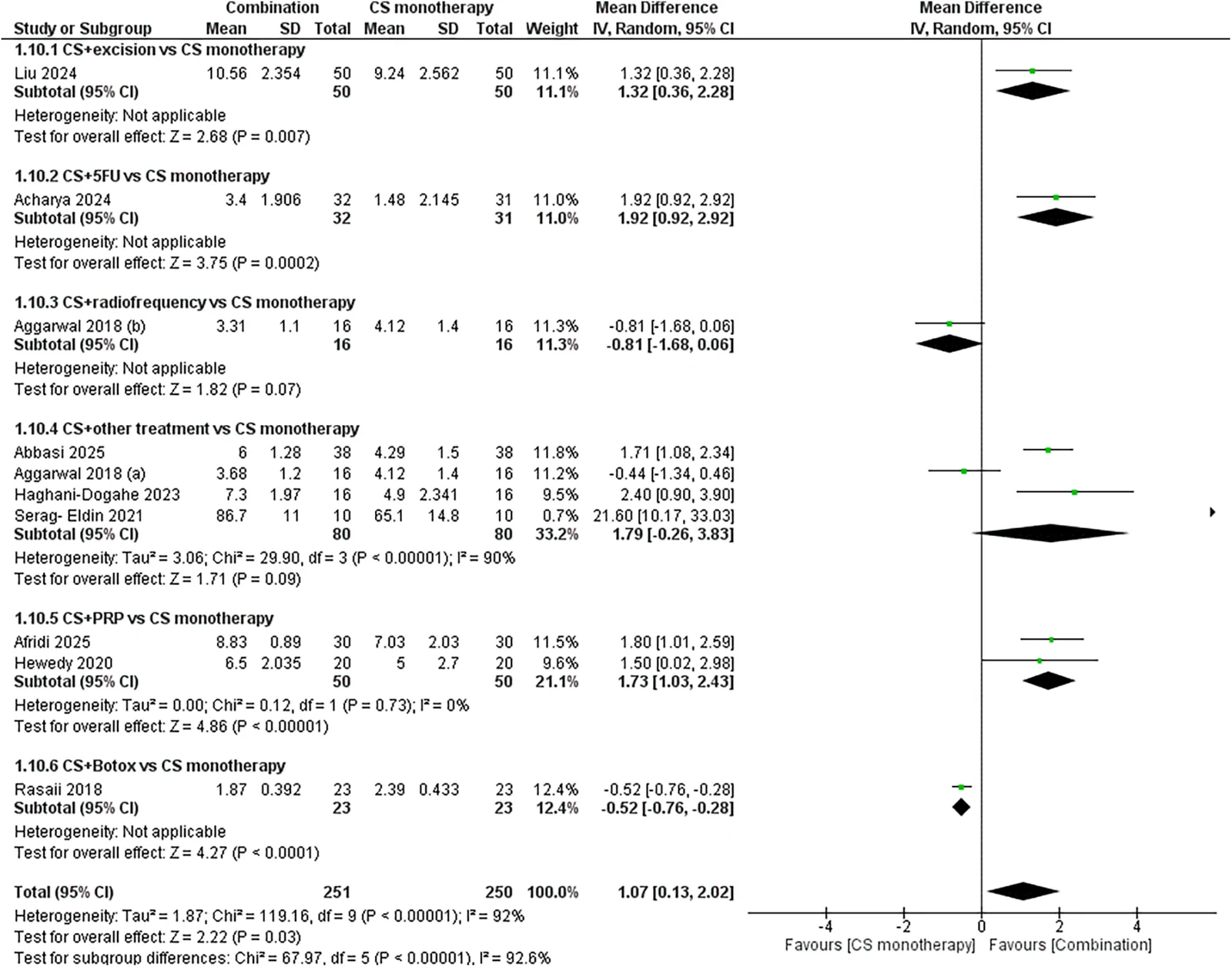
Comparison between combination therapy and monotherapy of intralesional corticosteroids regarding improvement in the VSS.

**Figure 10:**
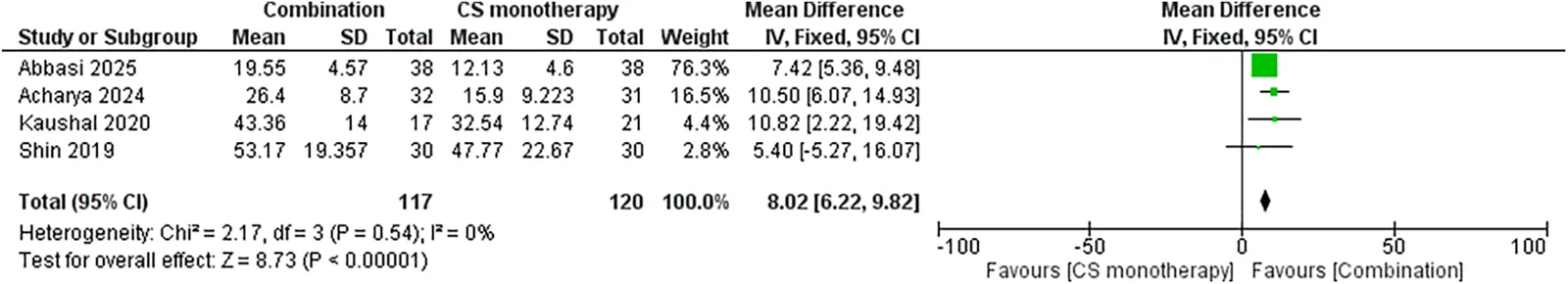
Comparison between combination therapy and monotherapy of intralesional corticosteroids regarding improvement in the POSAS.

The use of combination therapy was statistically significantly associated with a lower incidence of adverse events, with an OR of 0.36 (95% CI: 0.26, 0.49; *p* < 0.00001) and *I*^2^ = 71%, *p* < 0.0001. This difference was particularly evident in combinations containing 5-FU and platelet-rich plasma (PRP) (Figure 11).

**Figure 11:**
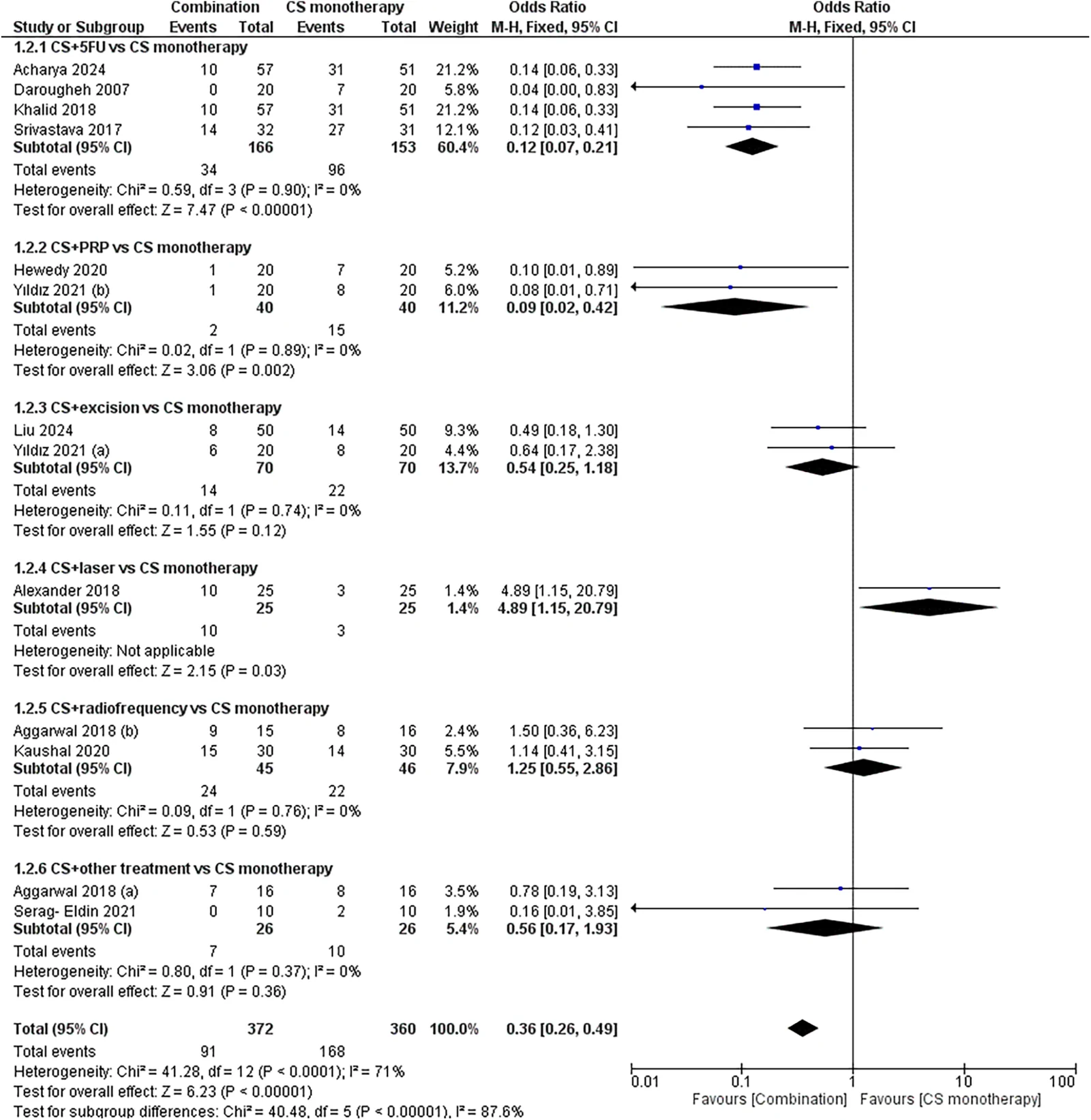
Comparison between combination therapy and monotherapy of intralesional corticosteroids regarding adverse events, sub-grouped by the therapy used in the combination.

The use of combination therapy was statistically significantly associated with a lower recurrence of keloids, with an OR of 0.33 (95% CI: 0.20, 0.53; *p* < 0.00001) and *I*^2^ = 35%, *p* = 0.17 (Figure 12).

**Figure 12:**
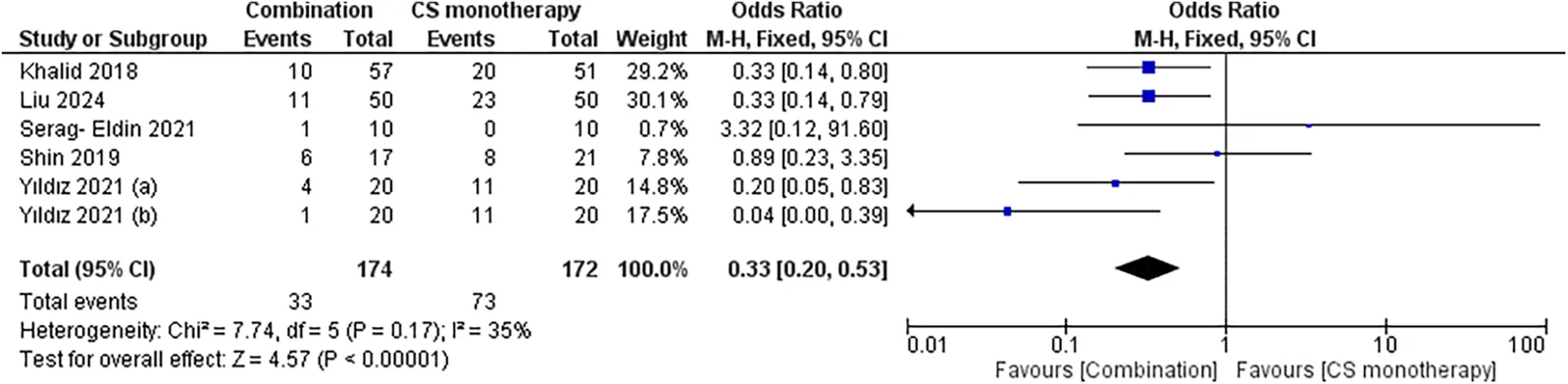
Comparison between combination therapy and monotherapy of intralesional corticosteroids regarding keloid recurrence.

#### 3.4.1 Subgroup analyses

For clinical efficacy, the pooled effect favored combination treatment over corticosteroids (CS) monotherapy for CS plus excision (OR: 3.16, 95% CI: 1.18–8.52; one study) and CS plus 5-fluorouracil (5-FU; OR: 4.45, 95% CI: 2.29–8.66; three studies). In contrast, the estimates for CS plus radiofrequency (OR: 1.27, 95% CI: 0.49–3.32; two studies) and CS plus other treatments (OR: 1.88, 95% CI: 0.81–4.36; two studies) were not statistically significant. There was no statistically significant evidence of a difference between efficacy subgroups (test for subgroup differences: *p* = 0.15).

For adverse events, pooled estimates favored combination treatment for CS plus 5-FU (OR: 0.12, 95% CI: 0.07–0.21; four studies) and CS plus PRP (OR: 0.09, 95% CI: 0.02–0.42; two studies). No statistically significant differences were detected for CS plus excision (OR: 0.54, 95% CI: 0.25–1.18; two studies), CS plus radiofrequency (OR: 1.25, 95% CI: 0.55–2.86; two studies), or CS plus other treatments (OR: 0.56, 95% CI: 0.17–1.93; two studies). In the single study of CS plus laser, the estimate favored CS monotherapy (OR: 4.89, 95% CI: 1.15–20.79). The test for subgroup differences was statistically significant (*p* < 0.0001), with substantial heterogeneity between subgroups (*I*^2^ = 87.6%).

For height, the pooled mean difference favored combination treatment in the CS plus 5-FU subgroup (MD: 0.81, 95% CI: 0.56–1.06; four studies). The estimates for CS plus PRP (MD: 1.40, 95% CI: −0.63 to 3.43; one study), CS plus Botox (MD: −0.17, 95% CI: −0.64 to 0.30; two studies), CS plus other treatments (MD: 0.61, 95% CI: −0.74 to 1.96; three studies), and CS plus radiofrequency (MD: 0.02, 95% CI: −0.18 to 0.22; one study) were not statistically significant. The test for subgroup differences was statistically significant (*p* < 0.0001; *I*^2^ = 85.9%). The clinical interpretation of these mean differences depends on the measurement units and the direction of scoring, which are not specified in the figure.

For VSS change, pooled estimates favored combination treatment for CS plus excision (MD: 1.32, 95% CI: 0.36–2.28; one study), CS plus 5-FU (MD: 1.92, 95% CI: 0.92–2.92; one study), and CS plus PRP (MD: 1.73, 95% CI: 1.03–2.43; two studies). The estimate for CS plus radiofrequency was not statistically significant (MD: −0.81, 95% CI: −1.68 to 0.06; one study). The estimate for CS plus other treatments was also not statistically significant (MD: 1.79, 95% CI: −0.26 to 3.83; four studies), while CS plus Botox favored monotherapy (MD: −0.52, 95% CI: −0.76 to −0.28; one study). The test for subgroup differences was statistically significant (*p* < 0.0001; *I*^2^ = 92.6%). These findings should be interpreted cautiously, given the small number of studies in several subgroups and substantial between-subgroup heterogeneity.

A subgroup analysis based on the study design was performed (Supplementary Figures S1–S7). Across the included studies, combination therapy was associated with fewer adverse events than monotherapy (OR: 0.35, 95% CI: 0.14–0.85; *p* = 0.023). Heterogeneity was substantial (*I*^2^ = 72.8%, *τ*^2^ = 1.173, *p* < 0.0001). The subgroup estimates were OR 0.26 (95% CI: 0.11–0.61) for RCTs and OR 1.31 (95% CI: 0.00–157,946,440.87) for non-RCTs. The difference between design subgroups was not statistically significant (*p* = 0.230). For height, the pooled mean difference favored combination therapy (MD: 1.14, 95% CI: 0.21–2.07; *p* = 0.021), with very high heterogeneity (*I*^2^ = 96.1%, *τ*^2^ = 1.468, *p* < 0.0001). The RCT subgroup estimate was MD 1.00 (95% CI: 0.13–1.87), while the non-RCT estimate was MD 4.60 (95% CI: 1.25–7.95); the subgroup difference was statistically significant (*p* = 0.039). For pain change, the overall pooled estimate favored combination therapy (MD: 0.66, 95% CI: 0.21–1.11; *p* = 0.011), with substantial heterogeneity (*I*^2^ = 69.5%, *τ*^2^ = 0.1615, *p* = 0.0032). The RCT subgroup estimate was MD 0.64 (95% CI: 0.13–1.16), and the non-RCT estimate was MD 1.10 (95% CI: −1.40 to 3.60). The subgroup difference was not statistically significant (*p* = 0.724). For the outcome labeled “itching change” in the plot, the overall effect was not statistically significant (MD: 1.56, 95% CI: −3.23 to 6.36; *p* = 0.376). Heterogeneity was moderate (*I*^2^ = 57.9%, *τ*^2^ = 3.1653, *p* = 0.0681). The RCT subgroup estimate was MD 3.93 (95% CI: −8.43 to 16.30), and the non-RCT estimate was MD 1.30 (95% CI: −0.87 to 3.47); there was no evidence of a subgroup difference (*p* = 0.393). For the POSAS change, the overall pooled mean difference favored combination therapy (MD: 8.02, 95% CI: 6.22–9.82; *p* < 0.0001). The RCT subgroup estimate was MD 8.10 (95% CI: 6.27–9.92), with no observed heterogeneity (*I*^2^ = 0%). The non-RCT study reported an MD of 5.40 (95% CI: −5.27 to 16.07). The subgroup difference was not statistically significant (*p* = 0.625). For recurrence, combination therapy was associated with a lower recurrence odds ratio than monotherapy (OR: 0.35, 95% CI: 0.13–0.91; *p* = 0.038). The overall heterogeneity was low and not statistically significant (*I*^2^ = 38.1%, *τ*^2^ = 0.056, *p* = 0.167). The RCT subgroup estimate was OR 0.36 (95% CI: 0.10–1.27), and the non-RCT estimate was OR 0.32 (95% CI: 0.00–12,424,247.15); the subgroup difference was not statistically significant (*p* = 0.916). For VSS change, the overall effect was not statistically significant (MD: 1.04, 95% CI: −0.39 to 2.46; *p* = 0.135), and heterogeneity was very high (*I*^2^ = 92.4%, *τ*^2^ = 1.4368, *p* < 0.0001). The RCT subgroup estimate was MD 0.95 (95% CI: −0.71 to 2.60), and the non-RCT estimate was MD 1.80 (95% CI: 1.01–2.59); no subgroup difference was detected (*p* = 0.309).

### 3.5 Certainty of evidence

The certainty of evidence varied across the evaluated outcomes and ranged from low to moderate according to the GRADE framework (Supplementary Table S2). No outcome was rated as high-certainty evidence. For clinical improvement, the certainty of evidence was rated as moderate. Combination therapy was associated with greater odds of clinical improvement compared with intralesional corticosteroid monotherapy (OR: 2.71, 95% CI: 1.80–4.09; *I*^2^ = 17%). The evidence was downgraded because of the risk of bias, the inclusion of mixed study designs, and limited treatment-specific evidence. For pain, the certainty of evidence was rated as low. The pooled mean difference was 0.67 (95% CI: 0.27–1.06; *I*^2^ = 69%), with downgrading primarily due to the risk of bias and inconsistency. For itching, the certainty of evidence was rated as low. The pooled standardized mean difference was 0.41 (95% CI: 0.06–0.77; *I*^2^ = 0%). Despite the absence of statistical heterogeneity, the certainty was limited by the small number of contributing studies and concerns regarding the risk of bias. For pigmentation, the certainty of evidence was rated as moderate. The pooled mean difference was 0.66 (95% CI: 0.40–0.92; *I*^2^ = 33%). The evidence was downgraded due to the risk of bias and the limited evidence base. For height/thickness, the certainty of evidence was rated as low. The pooled mean difference was 0.15 (95% CI: 0.06–0.23; *I*^2^ = 85%). The evidence was downgraded because of serious inconsistency and clinical heterogeneity among the contributing studies. For the VSS, the certainty of evidence was rated as low. Combination therapy was associated with greater improvement in VSS (MD: 1.07, 95% CI: 0.13–2.02). Statistical heterogeneity was not reported for this outcome in the manuscript. The certainty was downgraded because of the limited evidence base, risk of bias, and imprecision. For the POSAS, the certainty of evidence was rated as low. The pooled mean difference was 8.02 (95% CI: 6.22–9.82; *I*^2^ = 0%). The evidence was downgraded due to the limited number of contributing studies and the risk of bias. For adverse events, the certainty of evidence was rated as low. Combination therapy was associated with lower odds of adverse events (OR: 0.36, 95% CI: 0.26–0.49; I^2^ = 71%). The evidence was downgraded due to serious inconsistencies and differences among the evaluated adjunctive interventions. For recurrence, the certainty of evidence was rated as moderate. Combination therapy was associated with lower odds of recurrence (OR: 0.33, 95% CI: 0.20–0.53; I^2^ = 35%). The evidence was downgraded because of the risk of bias and variation in follow-up duration across the contributing studies. Overall, the GRADE assessment indicates that the observed benefits of combination therapy are supported predominantly by low- to moderate-certainty evidence. Therefore, although several pooled estimates favored combination therapy, the findings should be interpreted with cautious in view of methodological limitations, clinical heterogeneity, and the limited evidence available for several treatment-specific outcomes (Supplementary Table S2).

## 4 Discussion

### 4.1 Main findings

In the current study, the combination of intralesional corticosteroids with other therapies or drugs was more efficacious and safer than intralesional corticosteroids alone. This was observed as higher odds of clinical improvement and greater improvement in keloid characteristics, including pain, itching, pigmentation, and height. Moreover, the combination was associated with significant improvement in VSS and POSAS.

Regarding the safety parameters, the combination treatment was associated with decreased risk of adverse events and recurrence compared with intralesional corticosteroid alone. The findings reported above are focused on the overall effect of combinations; however, the sub-grouping showed that 5-FU combined with corticosteroids showed an obvious efficacy in these outcomes, but the sample size and the included previously published studies reporting the use of other combinations were limited, which fail to reach the power of statistical significance. Therefore, future RCTs are required to investigate the efficacy and safety of other combinations in addition to providing a comparison that demonstrates the best combination to be used in keloid treatment.

### 4.2 Clinical implications

Various modalities for managing keloids and hypertrophic scars have been documented, encompassing both surgical and non-surgical approaches, including surgical excision, laser therapy, radiation therapy, cryosurgery, occlusive dressings, and pharmacological agents such as verapamil, 5-FU, Botox, and silicone cream or gel application. However, a definitive therapeutic strategy that achieves universally accepted and permanent ablation of hypertrophic or keloid scars remains unestablished (36–38). The effectiveness of intralesional corticosteroid injections for individuals with keloids and hypertrophic scars is well established. Triamcinolone acetonide (TAC) is among the most frequently utilized corticosteroids. The dosage and treatment intervals range from 10 to 40 mg/mL, administered at intervals of 2–6 weeks for several months or until the scar has leveled (39, 40). Despite intralesional TAC demonstrating a 50%–100% success rate in several studies, the results have been unsatisfactory and have been accompanied by multiple adverse effects, including skin atrophy, telangiectasias, and changed pigmentation at the injection site (41, 42).

Regarding the use of surgical excision in combination with intralesional injection of TAC, the timing of using TAC is still debated. In the current meta-analysis, Liu et al. (25) reported that TAC was used preoperatively, while Yıldız et al. (18) reported that TAC was used intraoperatively or postoperatively. Also, previous studies (43, 44) showed different timings, which were mainly postoperative; however, the combination showed efficacy at any timing, as reported in the mentioned studies.

Concerning the medications utilized alongside TAC, 5-FU is a pyrimidine antimetabolite that inhibits thymidylate synthase, disrupts RNA synthesis, and thus impedes fibroblast activity. Furthermore, it has been observed to selectively inhibit collagen formation, potentially enhancing its efficacy in scar therapy (45). It has been revealed that intralesional injection of 5-FU once or twice weekly into hypertrophic scars and keloids is beneficial, resulting in decreased fibroblast activity in these lesions (36, 46). Intralesional 5-FU injection is safe and free of systemic effects when administered at a concentration of 50 mg/mL (39). However, adverse effects such as ulceration, erythema, and hyperpigmentation frequently occur with the use of pure 5-FU (47). To mitigate the adverse effects of the combination (TAC has been incorporated with 5-FU in a 1:9 ratio), a concentration of 4 mg/mL of TAC is not anticipated to contribute to efficacy; however, this minimal quantity of TAC likely serves a distinct function by alleviating potential inflammation induced by 5-FU (48, 49). In this context, intralesional TAC combined with 5-FU has been proposed as an alternative technique that may address the TAC treatment problem by enhancing scar quality while reducing the incidence of comorbidities.

Botulinum toxin is a neurotoxin composed of high-molecular-weight proteins, utilized for the treatment of blepharospasm, strabismus, hyperhidrosis, and facial wrinkles. Despite the existence of seven distinct serotypes of botulinum toxin in immunology, botulinum toxin type A is the most widely used clinical agent due to its paralytic properties. In recent years, the application of botulinum toxin for scar treatment has emerged, offering numerous advantages, such as diminishing muscle tone and inhibiting fibroblast activity. Botulinum toxin type A injection is acknowledged as a novel and efficacious therapy for keloids (50, 51). Botulinum toxin type A inhibits muscle and skin contractions adjacent to keloid tissue and alleviates tension throughout the wound healing process (52, 53). A separate study reported complete relief of intense pain and pruritus in keloids via the application of botulinum toxin type A (54). The alleviation of itching associated with botulinum toxin type A is believed to be linked to the activity of neuropeptides (55). Shaarawy et al. (56) showed that Botox-A may surpass TAC in enhancing scar pliability during medium-term follow-up. Further investigation is necessary to comprehensively ascertain the comparative efficacy of TAC and Botox-A vs. TAC alone.

Bleomycin has also been used to treat hypertrophic scars and keloids. Bleomycin was initially studied as a scar-reduction drug in the mid-1990s for its ability to suppress collagen synthesis in dermal fibroblasts by lowering stimulation by TGF-β1 (57). Bleomycin may surpass TAC in terms the POSAS; nonetheless, currently, the number of pertinent investigations is insufficient to draw a conclusive determination. We hypothesize that bleomycin could serve as a viable alternative treatment in the absence of TAC; however, further research is necessary to substantiate this claim.

In addition, PRP is an autologous platelet concentrate that promotes tissue repair, expedites wound healing, and alleviates pain and impairment in the affected region (58). According to the overall VSS score, Hewedy et al. (17) recently showed that TAC + PRP may yield considerably superior outcomes compared to TAC alone in keloid treatment. The current meta-analysis indicated a reduced incidence of adverse events with this combination compared with TAC alone.

Verapamil, a calcium channel blocker, exhibits anti-fibrotic properties that demonstrate potential efficacy in keloids and hypertrophic scars. Verapamil seems to diminish the extracellular matrix by inhibiting collagen synthesis. Furthermore, verapamil may suppress inflammatory responses by inhibiting platelet aggregation and diminishing neutrophil activity (59). Verapamil increases procollagenase synthesis and reduces collagen creation in scars, subsequently leading to the depolymerization of actin filaments and a decrease in fibrous tissue production (60).

Previous studies indicate that TAC frequently results in undesirable consequences, such as skin atrophy and telangiectasia, in up to 63% of patients (61). Zhuang et al. (62) found that patients administered TAC (20 or 40 mg/mL) injections exhibited a higher propensity for skin atrophy compared to those treated with 5-FU or verapamil. At a TAC concentration of 10 mg/mL, TAC therapy did not differ significantly from Botox-A or bleomycin in the occurrence of skin atrophy. Telangiectasia has been detected more frequently in patients undergoing TAC treatment than in those treated with 5-FU, bleomycin, or TAC + 5-FU (62). According to current research and the findings of this study, we contend that side effects, such as skin atrophy or telangiectasias, may occur with TAC monotherapy and be significant. Certain patients may be unresponsive to TAC injections, and among those who do respond, the efficacy of TAC may vary between individuals and across therapy phases. These observations indicate that prompt identification of non-responding individuals is essential for enhancing treatment outcomes. Additional research is required to validate the efficacy of TAC across various patient populations.

The effectiveness of corticosteroid injections for keloid and hypertrophic scars is now well established, with the predominant regimen involving TAC injections of 10–40 mg/mL administered at intervals of 1–6 weeks for several months, continuing until the scar is flattened (39, 63).

The findings have several practical clinical implications. The overall analysis supports the potential benefit of adding an adjunctive treatment to intralesional corticosteroid therapy compared with corticosteroid monotherapy. However, adjunctive modalities should not be considered interchangeable, as they differ substantially in their mechanisms of action, treatment protocols, and available evidence.

Among the evaluated combinations, 5-FU-containing corticosteroid regimens demonstrated the most consistent evidence of benefit across the outcomes assessed in this review. This finding suggests that corticosteroid plus 5-FU may currently represent the most evidence-supported adjunctive strategy among the treatments evaluated. This interpretation is also consistent with previous systematic reviews evaluating corticosteroid plus 5-FU, which have reported greater treatment effectiveness and improvements in scar height compared with corticosteroid monotherapy.

However, the findings should not be interpreted as establishing that 5-FU is universally superior to every other adjunctive treatment. Several other modalities, including PRP, verapamil, botulinum toxin, laser therapy, cryotherapy, excision, radiofrequency, pentoxifylline, onion extract, and hyaluronidase, were represented by fewer studies or demonstrated less consistent evidence in the present analysis. Consequently, the evidence for these approaches remains insufficient to establish their comparative effectiveness or to determine whether they provide clinically meaningful advantages over 5-FU-containing combinations or corticosteroid monotherapy.

The limited evidence for several adjunctive therapies is particularly important when interpreting the absence of significant differences in some subgroup analyses. A lack of statistical significance should not necessarily be interpreted as evidence of equivalent efficacy, because several treatment-specific comparisons were based on few studies and participants. Further adequately powered randomized controlled trials are therefore required, particularly for less-well-studied adjunctive modalities. Future studies should use standardized corticosteroid and adjunctive treatment doses, clearly defined treatment intervals, validated outcome measures, and sufficiently long follow-up to permit reliable assessment of both treatment response and recurrence.

From a practical clinical perspective, the current evidence therefore supports consideration of corticosteroid-based combination therapy, with 5-FU-containing regimens having the strongest evidence within the present review. Nevertheless, treatment selection should remain individualized based on lesion characteristics, anatomical site, severity, previous treatment response, adverse-effect profile, and availability of the treatment. The available evidence does not currently justify recommending a single combination regimen for all patients.

### 4.3 Comparison with literature

In accordance with the present findings, Jiang et al. (64) reported that the combination of TAC + 5-FU was associated with better clinical efficacy and fewer complications compared with TAC only. A meta-analysis was conducted by Kuang et al. (65) to compare the efficacy and safety of verapamil and TAC in the treatment of keloids and hypertrophic scars. This meta-analysis showed that TAC had a better therapeutic efficacy than verapamil, while verapamil was safer. This shows the association of TAC with a high risk of adverse effects that necessitate the use of a combination drug with it to lower its dose and the resulting complications.

### 4.4 Strengths and limitations

This systematic review and meta-analysis compared all available combinations containing intralesional corticosteroids with intralesional corticosteroids alone across different efficacy and safety outcomes. Although we included a large sample size obtained from 20 studies, most combination therapies did n't have enough data to yield a significant outcome during the meta-analysis. Although restricting the analysis to RCTs could theoretically assess the influence of study design, such a sensitivity analysis was not considered sufficiently informative in the present evidence base because the number of eligible studies was already limited, and further exclusion of non-randomized studies and cohort studies would have substantially reduced the available statistical information and potentially resulted in unstable or non-estimable estimates. Nevertheless, the absence of an RCT-only sensitivity analysis is an important limitation, as it restricts our ability to assess the robustness of the pooled estimates to study design. In particular, because non-randomized and observational studies contributed to the evidence base, we cannot exclude the possibility that study design influenced the magnitude or direction of the pooled treatment effects. Accordingly, the overall estimates should be interpreted cautiously and should not be considered equivalent to estimates derived exclusively from randomized evidence.

The overall pooling of adjunctive therapies should be interpreted in the context of the primary clinical question of this review: whether adding an adjunctive treatment to intralesional corticosteroids provides additional benefit compared with corticosteroid monotherapy. The included adjunctive therapies differ substantially in their biological mechanisms and treatment characteristics; therefore, the overall estimate should not be interpreted as evidence that all combination regimens are equally effective. To address this heterogeneity, treatment-specific subgroup analyses were performed whenever sufficient data were available, and the effect estimates for individual adjunctive therapies are reported. These analyses suggested that the magnitude and consistency of benefit varied between treatment modalities, with 5-FU-containing combinations demonstrating a particularly consistent signal of benefit across several outcomes. For outcomes in which individual treatment categories were represented by very few studies, subgroup analysis was not considered sufficiently informative because of the limited statistical information and resulting imprecision. Thus, the overall pooled estimate provides evidence regarding the broader strategy of adjunctive treatment, while the subgroup analyses provide more clinically specific information where the available evidence permitted. The substantial heterogeneity observed for several outcomes limits the precision with which the pooled estimates can be generalized across different clinical settings. In particular, variations in corticosteroid dosing and treatment schedules, adjunctive treatment protocols, lesion characteristics, anatomical location, treatment intervals, and follow-up duration may have contributed to differences in treatment effects. Consequently, the pooled estimates for outcomes with substantial heterogeneity should be interpreted as average effects across heterogeneous clinical settings rather than as universally applicable treatment effects. Publication bias could not be reliably assessed for most outcomes because the number of studies contributing to the individual meta-analyses was limited.

### 4.5 Recommendations

Based on the current evidence, adjunctive therapy combined with intralesional corticosteroids may be considered in selected patients, rather than being universally preferred over intralesional corticosteroid monotherapy. Among the adjunctive approaches evaluated, 5-FU-containing regimens demonstrated the most consistent evidence of benefit; however, the available evidence is insufficient to establish a single combination regimen as superior or appropriate for all patients. Treatment selection should therefore be individualized based on lesion characteristics, anatomical location, prior treatment response, anticipated adverse effects, and treatment availability. Further adequately powered RCTs directly comparing individual combination regimens with corticosteroid monotherapy are required using standardized treatment protocols, validated outcome definitions, and sufficiently long follow-up to determine the comparative effectiveness and safety of specific adjunctive therapies.

In this systematic review and meta-analysis, adjunctive treatment combined with intralesional corticosteroids was associated with favorable outcomes compared with corticosteroid monotherapy across several clinical and scar-related measures. However, the considerable clinical and methodological heterogeneity among the included studies, differences in study designs and treatment protocols, relatively limited sample sizes for several adjunctive modalities, and variation in follow-up duration reduce the certainty and generalizability of these findings. Therefore, the available evidence does not support a universal recommendation that combination therapy should be preferred over corticosteroid monotherapy for all patients. Among the evaluated adjunctive approaches, 5-FU-containing combinations demonstrated the most consistent evidence of benefit in the present review, whereas the evidence for several other modalities remains limited. Further adequately powered randomized controlled trials using standardized treatment protocols, validated outcome measures, and sufficiently long follow-up are needed to determine the comparative effectiveness and optimal use of individual adjunctive therapies.

## Data Availability

The original contributions presented in the study are included in the article/Supplementary Material, further inquiries can be directed to the corresponding author.
