# Supplementary material for: Efficacy and safety of intralesional corticosteroids alone or in combination with other treatments for the management of keloids: a systematic review and meta-analysis
Source: Front Med (Lausanne). 2026 Oct 26;13:1930913. doi: 10.3389/fmed.2026.1930913 (PMC13746366; doi:10.3389/fmed.2026.1930913)
Supplement: Supplementary file 1 [file Supplementaryfile1.docx]

Table S1: Search strategy for each database

| Database | Date range | Reconstructed search strategy based strictly on the submitted manuscript |
| --- | --- | --- |
| PubMed | Inception–May 2026 | ("Keloid"[Title/Abstract]  OR "Keloids"[Title/Abstract]  OR "Keloid Scar"[Title/Abstract]  OR "Keloid Scars"[Title/Abstract])  AND  ("Intralesional"[Title/Abstract]  OR "Intralesional Injection"[Title/Abstract]  OR "Intralesional Injections"[Title/Abstract])  AND  ("Corticosteroid"[Title/Abstract]  OR "Corticosteroids"[Title/Abstract]  OR "Triamcinolone"[Title/Abstract]  OR "Triamcinolone Acetonide"[Title/Abstract]  OR "TAC"[Title/Abstract]) |
| Scopus | Inception–May 2026 | ABS(keloid OR keloids OR "keloid scar" OR "keloid scars")  AND  ABS(  intralesional OR "intralesional injection" OR "intralesional injections")  AND  ABS(corticosteroid OR corticosteroids OR triamcinolone OR "triamcinolone acetonide" OR TAC) |
| Web of Science | Inception–May 2026 | TITLE-ABS(keloid OR keloids OR "keloid scar" OR "keloid scars")  AND  TITLE-ABS(  intralesional OR "intralesional injection" OR "intralesional injections")  AND  TITLE-ABS(  corticosteroid OR corticosteroids OR triamcinolone OR "triamcinolone acetonide" OR TAC) |

Table S2: GRADE Summary of Findings table

| **Outcome** | **Effect estimate** | **Heterogeneity** | **Certainty of evidence** | **Main reasons for downgrading** |
| --- | --- | --- | --- | --- |
| **Clinical improvement** | OR 2.71 (95% CI 1.80–4.09) | I² = 17% | **Moderate** | Risk of bias; mixed study designs |
| **Pain** | MD 0.67 (95% CI 0.27–1.06) | I² = 69% | **Low** | Risk of bias; inconsistency |
| **Itching** | SMD 0.41 (95% CI 0.06–0.77) | I² = 0% | **Low** | Limited number of contributing studies; risk of bias |
| **Pigmentation** | MD 0.66 (95% CI 0.40–0.92) | I² = 33% | **Moderate** | Risk of bias; limited evidence |
| **Height/thickness** | MD 0.15 (95% CI 0.06–0.23) | I² = 85% | **Low** | Serious inconsistency; clinical heterogeneity |
| **VSS** | MD 1.07 (95% CI 0.13–2.02) | NR in manuscript | **Low** | Limited evidence; risk of bias; imprecision |
| **POSAS** | MD 8.02 (95% CI 6.22–9.82) | I² = 0% | **Low** | Limited number of studies; risk of bias |
| **Adverse events** | OR 0.36 (95% CI 0.26–0.49) | I² = 71% | **Low** | Serious inconsistency; differences in interventions |
| **Recurrence** | OR 0.33 (95% CI 0.20–0.53) | I² = 35% | **Moderate** | Risk of bias; varying follow-up |

CI, confidence interval; GRADE, Grading of Recommendations Assessment, Development and Evaluation; I², I-squared statistic; MD, mean difference; OR, odds ratio; POSAS, Patient and Observer Scar Assessment Scale; SMD, standardized mean difference; VSS, Vancouver Scar Scale

Figure S1. Subgroup analysis for adverse events based on the study design

Figure S2. Subgroup analysis for recurrence rates based on the study design

Figure S3. Subgroup analysis for pain change based on the study design

Figure S4. Subgroup analysis for itching change based on the study design

Figure S5. Subgroup analysis for height changes based on the study design

Figure S6. Subgroup analysis for POSAS changes based on the study design

Figure S7. Subgroup analysis for VSS changes based on the study design

Table S3. Studies' contribution to evidence

| **Outcome** | **Studies;** | **participants** |
| --- | --- | --- |
| Clinical efficacy | 8 | 531 |
| Adverse events | 13 | 732 |
| Recurrence | 6 | 346 |
| Pain change | 7 | 237 |
| Itching change | 4 | 127 |
| Pigmentation change | 2 | 72 |
| Height | 11 | 491 |
| VSS change | 10 | 501 |
| POSAS change | 4 | 237 |
